# Fontan-associated liver disease: the importance of multidisciplinary teamwork in its management

**DOI:** 10.3389/fmed.2024.1354857

**Published:** 2024-11-27

**Authors:** Tomoya Tsuchihashi, Yuki Cho, Daisuke Tokuhara

**Affiliations:** ^1^Department of Pediatrics, Wakayama Medical University, Wakayama, Japan; ^2^Department of Pediatrics, Osaka Metropolitan University, Osaka, Japan

**Keywords:** Fontan procedure, Fontan-associated liver disease, liver fibrosis, hepatocellular carcinoma, gastroesophageal varix, protein-losing enteropathy, liver stiffness

## Abstract

The Fontan operation, which directly connects the superior and inferior vena cava to the pulmonary artery, is a palliative surgery for children with a functional or anatomic single ventricle. This procedure leads to hemodynamic changes (Fontan circulation) in patients, who tend to develop congestive hepatic fibrosis characterized by sinusoidal fibrosis and dilatation beginning approximately 10 years after the procedure. In addition, in the context of severe fibrosis and cirrhosis, hepato-gastrointestinal complications including hepatocellular carcinoma, focal nodular hyperplasia, and portal hypertension can arise. Fontan-associated liver disease (FALD) encompasses the broad spectrum of liver alterations secondary to postoperative hemodynamic changes, and the effective management of FALD requires contributions from specialists in hepatology, gastroenterology, surgery, radiology, histopathology, and pediatric and adult cardiology. In this article, we outline the pathogenesis of FALD and discuss the importance of a multidisciplinary collaborative approach to its management.

## 1 Introduction

**Figure 1:**
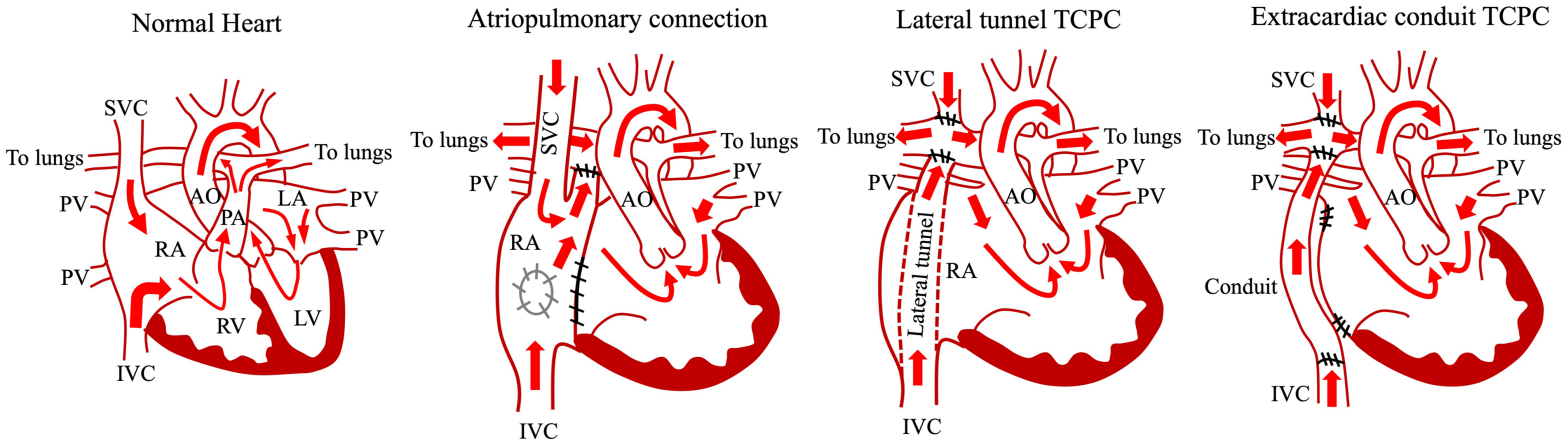
Common types of Fontan procedure. AO, aorta; IVC, inferior vena cava; LA, left atrium; LV, left ventricle; PA, pulmonary artery; PV, pulmonary vein; RA, right atrium; RV, right ventricle; SVC, superior vena cava; TCPC, total cavopulmonary connection.

**Figure 2:**
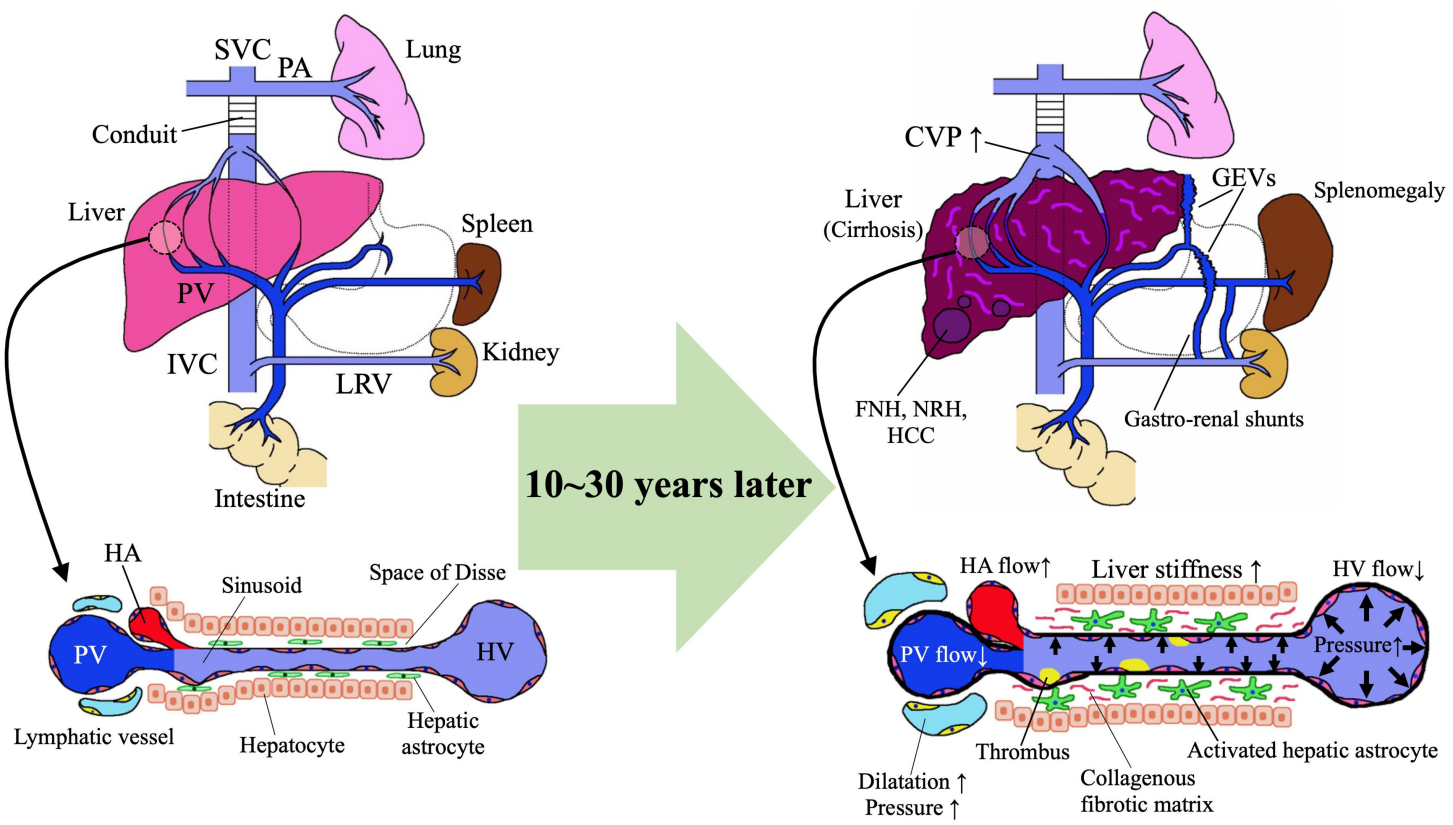
Pathomechanism of Fontan-associated liver disease (FALD). FNH, focal nodular hyperplasia; GEVs, gastroesophageal varices; HA, hepatic artery; HCC, hepatocellular carcinoma; HV, hepatic vein; IVC, inferior vena cava; LRV, left renal vein; NRH, nodular regenerative hyperplasia; PA, pulmonary artery; PV, portal vein; SVC, superior vena cava.

The Fontan operation, which connects the superior vena cava (SVC) and the inferior vena cava (IVC) to the pulmonary artery via a conduit or intra-arterial baffle, is a palliative surgery for patients with complex congenital heart disease (e.g., single ventricle disease) (Figure 1) (1). The postoperative mortality associated with the Fontan procedure has decreased significantly over the past few decades due to improvements in surgical techniques and perioperative management (2). The 10-, 20-, and 30-year survival rates for patients undergoing Fontan operation in the 1970s and 1980s were 69, 57, and 39%, respectively, but since 2001, the 10-year survival rate has improved to 95% (3). Given the increased survival rates, the number of patients with Fontan circulation is anticipated to increase—from 66 per million in 2020 to an estimated 79 per million in 2030—with a corresponding increase in the proportion of adult Fontan patients from 55 to 64% (4–6). Consequently, many post-Fontan patients with complex backgrounds will need to be transferred from pediatric to adult cardiology services. In addition, prolonged exposure to the unique environment of Fontan circulation causes a variety of extracardiac complications (e.g., thrombosis and hemorrhage, protein-losing enteropathy, cirrhosis, nephropathy), which diminish long-term quality of life after the Fontan procedure (7–9). In particular, hepato-gastrointestinal complications (e.g., cirrhosis, hepatocellular carcinoma [HCC], and gastroesophageal varices [GEVs]) late after Fontan surgery were poorly recognized initially but are closely associated with patient survival (Figure 2). Therefore, early detection and therapeutic intervention of hepato-gastrointestinal complications is essential to improve the survival rate and quality of life of patients who have undergone the Fontan procedure. In this article, we outline the pathogenesis and management of hepato-gastrointestinal complications of Fontan surgery, with a focus on Fontan-associated liver disease (FALD), and discuss the importance of a multidisciplinary care team that includes pediatric and adult cardiologists, pediatric and adult gastroenterologists and liver specialists, imaging and interventional radiologists, thoracic surgeons, histopathologists, psychiatrists, and liver and heart transplant surgeons.

## 2 Fontan circulation

The Fontan operation is the definitive palliative procedure for patients with complex congenital heart disease in whom biventricular repair is difficult (Figure 1) (1, 10, 11). The Fontan operation is generally performed when patients are around 3 years old (12–14), and the systemic circulation is maintained by the single ventricle and a vein is connected directly to the pulmonary artery instead of the pulmonary ventricle, which reduces the load on the single ventricle and decreases cyanosis by maintaining pulmonary circulation with venous pressure (15–17). Since 1968, when the procedure was first performed by the French surgeon Francis Fontan for the tricuspid atresia (1), this surgical procedure has developed by several technical modifications (Figure 1). In the atriopulmonary connection (APC), the right atrium is connected to the right pulmonary artery, whereas the atrial septal defect and the hypoplastic tricuspid valve are closed (18, 19). Because of the complications (e.g., massive dilatation of the right atrium, atrial tachyarrhythmias) occurred after APC, the procedure was modified to the lateral tunnel total cavopulmonary connection (TCPC) in which the right atrium was baffled with an intraatrial patch and SVC and IVC were redirected to the right pulmonary artery (15, 20) (Figure 1). A bidirectional Glenn performed at an earlier age as an intermediate step before the Fontan procedure decreased total mortality and morbidity by Fontan circulation (21). Lateral tunnel TCPC contributed to reduce arrhythmic risk and energy loss in the system. At the end of the 1980s, the extracardiac TCPC, the most recent modification, was developed in which the intra-atrial routing of the venous flow was replaced by the insertion of an extracardiac conduit between the inferior vena cava and the right pulmonary artery (22, 23) (Figure 1). This procedure further contributed to reduce complications (e.g., arrhythmic risk) (24). In addition to those technical modifications, advances in perioperative management techniques have significantly improved the short- and medium-term survival rates after Fontan surgery (25–27). However, due to Fontan-associated complications, the long-term postoperative survival rate for the Fontan procedure remains low compared with those for other congenital heart diseases (28, 29). Anatomic and functional issues (elevated central venous pressure [CVP], Fontan track stenoses, AV valve regurgitation, poor pulmonary arterial compliance, thromboses, single ventricle systolic and/or diastolic dysfunction) arise from the Fontan procedure and circulation are involved in the pathomechanisms of FALD. In terms of the CVP, to maintain a sufficient cardiac output for the systemic circulation, the heart must have a pumping function, a low afterload, and an adequate preload. In the normal heart, increased right ventricular pressure connected to the pulmonary artery increases pulmonary blood flow and provides sufficient preload to the left ventricle. CVP is generally low because the central veins are protected from the right ventricular pressure by the tricuspid valve (30). In contrast, it is necessary to have equal or higher CVP (the range of 12 to 14 mmHg) than the pulmonary pressure in order to recruit the whole pulmonary vascular bed (31). At the same time, the CVP should be low in order to prevent lymphatic stasis and edema leading to protein-losing enteropathy. However, the pulmonary blood flow produced by the central veins is limited and cannot provide sufficient left ventricular preload. This prolonged state of elevated CVP and low cardiac output is the underlying cause of the multiorgan damage currently faced by Fontan patients. Furthermore, in the Fontan circulation, increased CVP and increased vascular resistance due to the artificial vessels cause poor pulmonary venous return and increased afterload. In addition, stenosis or abnormal connections of the pulmonary vessels, as well as reduced thoracic compliance, alveolar cell damage, and increased collateral blood vessels due to open chest surgery, can increase pulmonary vascular resistance (32, 33). Also, underdevelopment of the pulmonary vessels and the presence of fenestration lead to ventilation–perfusion imbalance and reduce oxygen saturation in the organs (34, 35). Furthermore, patients requiring a Fontan operation often have concomitant non-cardiac congenital anomalies, such as chromosomal abnormalities, as well as induced muscle weakness due to exercise limitation during their medical management, thereby markedly deranging the organ environment. In particular, the peripheral musculature, venous capacitance, and tone have a significant influence on venous return flow, and it has been reported that appropriate training of the lower limbs as well as cardiopulmonary training can directly influence cardiac function in the Fontan circulation (36). As a result of these hemodynamics after Fontan procedure, Fontan patients develop multi-organ changes and diseases sooner than do healthy people, leading to the need for specialized multi-organ follow-up from a young age (37–39).

## 3 Definition and histological features of FALD

Although a standard definition of FALD has not been established, the European Association for the Study of the Liver (EASL) and the European Reference Network on Rare Liver Diseases (ERN-RARE-LIVER) stated in a 2023 position paper that “FALD encompasses a broad spectrum of structural, functional, and clinical liver alterations secondary to Fontan hemodynamic changes” (40). Hepato-gastrointestinal complications, such as liver fibrosis, cirrhosis, and HCC, as well as GEVs and protein-losing enteropathy (PLE) are just some of the potential components of FALD (Figure 2) (41, 42). Due to differences in assessment method and time of analysis since surgery, the reported frequency of hepato-gastrointestinal complications in FALD varies: 0.85–58.3% for liver fibrosis (mild to severe and cirrhosis) (43–48); 1.15–9.8% for HCC (47, 49–51); 14.8–33.8% for GEVs (42, 45, 52); and 2.5–41.6% for PLE (45, 53–57). FALD is often clinically asymptomatic and is suggested through regular imaging or bloodwork or is diagnosed through liver biopsy during long-term follow-up. In addition, regular imaging sessions frequently reveal hepatomegaly, which reportedly is associated with decreased cardiovascular prognosis in adolescent and young adult patients after Fontan surgery (58, 59). Splenomegaly and jaundice are also frequent clinical features of FALD that are indicated through imaging and laboratory examinations (42, 59). In advanced FALD, portal hypertension due to liver cirrhosis can lead to symptoms of ascites, hepatic encephalopathy, and variceal hemorrhage.

Given this diversity of symptoms, it is crucial to remember that FALD is universal to patients with Fontan circulation although it manifests to various degrees from individual to individual. Among the broad spectrum of liver alterations in FALD, the most characteristic are its histologic features, which differ from those of other chronic liver diseases (e.g., hepatitis B [HBV], hepatitis C [HCV], and non-alcoholic fatty liver disease [NAFLD]) (41, 43, 44, 60). The histologic alterations in FALD are characterized by fibrosis, mainly in the central venous and sinusoidal regions, and dilatation of the sinusoids, reflective of hepatic congestion (Figures 2a,b) (41, 43, 60). As fibrosis progresses, bridging fibrosis and fibrous septa form (Figures 2c,d), ultimately resulting in cirrhosis. In contrast, infiltration of the liver by inflammatory cells occurs only rarely in FALD (41).

**Figure 3:**
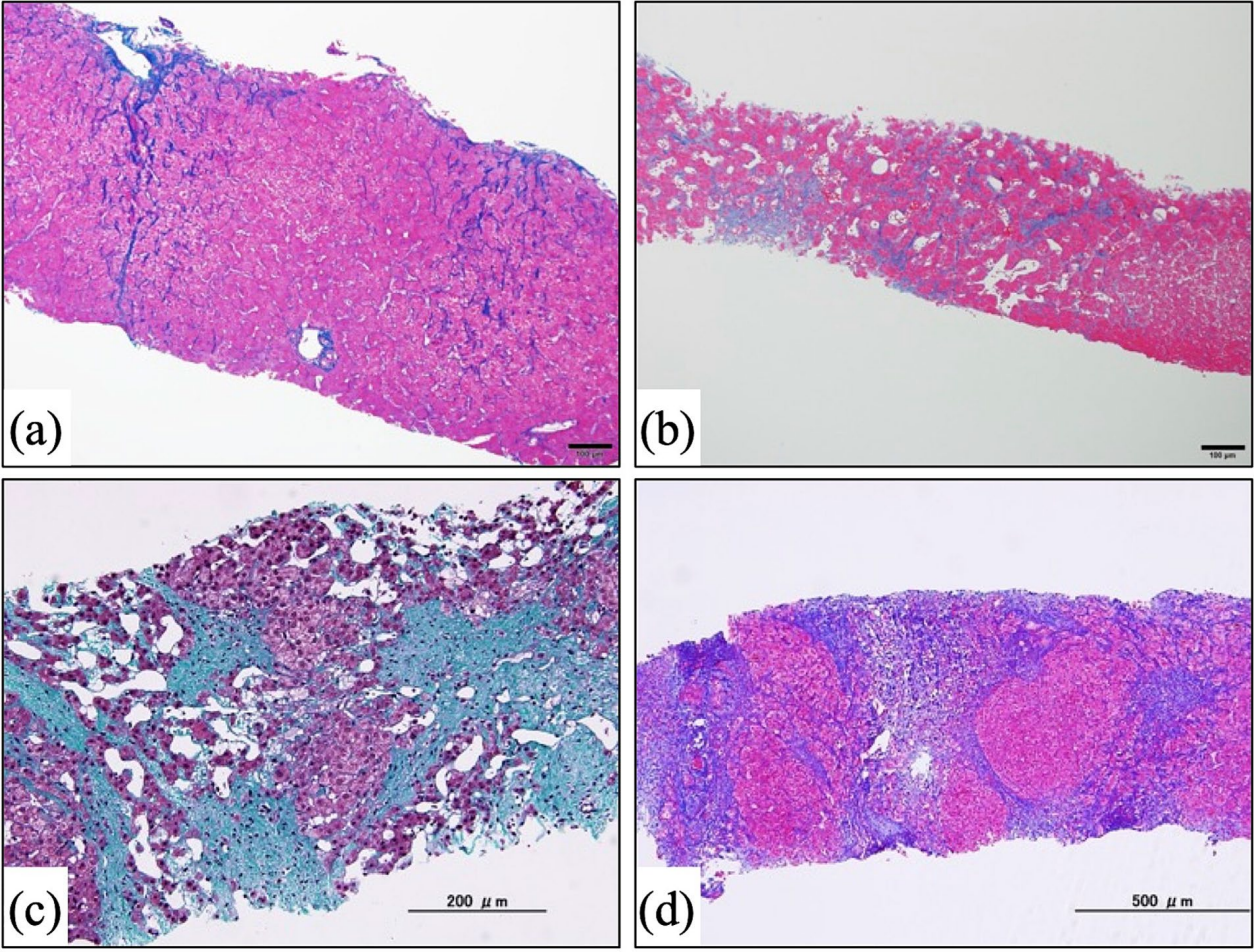
Microscopic images of liver biopsy specimens. Specimens were taken from post-Fontan patients in our previous study (41). Liver fibrosis was evaluated according to the congestive hepatic fibrosis score (CHFS) (60). (a) Azan staining. This patient underwent Fontan surgery 12.2 years earlier. CHFS 2A. (b) Azan staining. A patient who had the Fontan procedure 14.1 years previously. CHFS 2B. (c) Masson trichrome staining. This patient received the Fontan operation 18.8 years earlier. CHFS 3. (d) Azan staining. A patient who had the Fontan procedure 17.7 years previously. CHFS 3.

Several studies have clarified that almost all patients who have undergone Fontan surgery have some degree of characteristic sinusoidal fibrosis with or without sinusoidal dilatation (Figures 3a–d), given the propensity of Fontan circulation to precipitate liver damage (41, 61–64). This hepatic fibrosis is not only induced postoperatively: several studies suggest that, due to the cumulative effects of surgery, cyanosis, and other factors, liver damage is present either before or occurs soon after Fontan reconstruction (65, 66). The estimated morbidity of FALD varies among studies, possibly due to differences in analytic methods (e.g., liver biopsy, computed tomography [CT], magnetic resonance imaging [MRI]) and patient age or time since surgery. For example, liver biopsies obtained from 67 patients at a mean of 17.3 years postoperatively revealed a significant correlation between time since surgery and liver fibrosis (64). Liver biopsies taken from 13 patients postoperatively (mean, 16.9 years) showed that all 13 patients had fibrosis primarily in the sinusoidal region, and 3 (23%) had cirrhosis (43). Liver biopsy or imaging (CT and MRI) of 195 patients (mean, 23.4 years postoperatively) indicated cirrhosis in 20.5% (46). In our own study, all 22 patients evaluated (mean, 13.7 years postoperative) had at least mild sinusoidal fibrosis (41).

## 4 Pathophysiology of liver fibrogenesis of FALD

Hepatic astrocytes in the sinusoidal Disse space play a central role in liver fibrogenesis after the Fontan procedure (Figure 2). The increased CVP and reduced cardiac output after Fontan surgery cause hepatic congestion associated with reduced hepatic venous blood flow (67), sinusoidal dilation, and increased sinusoidal pressure due to reduced portal venous blood flow and portal hypertension (68), which culminate in intrahepatic hypoxia, thrombus formation, and elevated sinusoidal stiffness (Figure 2) (69–71). These factors activate hepatic astrocytes and thus accelerate the production of extracellular matrix proteins, such as collagen, with the development of liver fibrosis (69). A study using rat cells disclosed that increased sinusoidal tissue stiffness causes hepatic astrocytes to differentiate into myofibroblast-like cells that produce collagen in the absence of transforming growth factor *β* (70). In a murine study in which liver congestion was induced through partial ligation of the inferior vena cava, lipopolysaccharide was transported via the portal vein from the intestinal tract to the liver and promoted the production of sphingosine-1-phosphate via capillarization of hepatic sinusoidal endothelial cells followed by the development of liver fibrosis (72). In the hemodynamics after the Fontan operation, the hepatic venous blood flow enters directly into the lungs, thus making the liver susceptible to the effects of increased CVP. The poor cardiac output due to the limited preload in children with single ventricles further elevates CVP after the Fontan operation. These increases in CVP are transmitted to the hepatic veins and then to the sinusoids, thereby dilating sinusoids, increasing sinusoidal stiffness, and promoting liver fibrosis. Together with liver hypoxia due to fenestration and arteriovenous shunts, the increased CVP and reduced cardiac output decrease first portal venous blood flow and then the oxygen saturation concentration of the portal blood. Hepatic hypoxia activates astrocytes to produce vascular endothelial growth factor and type I collagen, which are exacerbating factors for liver fibrosis. Stasis of hepatic inflow due to the increased CVP promotes the formation of intrahepatic microthrombi, which further stimulate hepatic astrocytes and thus exacerbate hepatic fibrosis (71).

## 5 HCC

Given the context of severe liver fibrosis and cirrhosis, HCC develops in post-Fontan patients. Among 122 patients with FALD, 9.8% developed HCC at a median age of 30–32.5 years, and the incidence of HCC at 10, 20, and 30 years postoperatively was 0.8, 2.9, and 13.3%, respectively (51). HCC was significantly more common in FALD patients with cirrhosis (58.3%) than in non-cirrhotic patients (17.3%) (51). The comorbidity of FALD with HCC results in a poor prognosis, with a reported 12-month survival rate of 50–53% and a 24-month survival rate of 37–40% (73, 74). Lung metastases are the most frequent extrahepatic metastases of HCC (75). A systematic review of 65 Fontan-associated cases of biopsy-confirmed postoperative HCC revealed that complications due to HCC typically emerged at least 10 years after surgery (age range, 12–52 years); only 1 of the 65 cases reviewed occurred earlier than 10 years postoperatively. In addition, approximately 70% of the HCC cases had a cirrhotic background; however, HCC can occur in the absence of cirrhosis (76). Therefore, compared with adolescent and adult patients who underwent a Fontan procedure as children, pediatric postoperative patients typically do not experience HCC (76) but instead often show benign focal liver lesions, including focal nodular hyperplasia (FNH) and nodular regenerative hyperplasia (NRH). Therefore, accurate differentiation of HCC from other benign focal liver lesions is necessary for effective clinical management of FALD.

## 6 GEVs

GEVs can occur as a result of portal hypertension due to advanced liver fibrosis and cirrhosis in patients after Fontan procedure (45). GEVs can cause life-threatening events (e.g., rupture and bleeding) in Fontan postoperative patients (57, 77). In one report, GEVs and portal hypertension were present in 19.2 and 36%, respectively, of Fontan patients at a mean of 17 years postoperatively (52). In another study, GEVs were associated with cirrhosis in 4 (33.3%) of 12 patients (mean, 14.1 years postoperative) (45). In our own study, 4 (14.8%) of 27 patients (mean, 12.9 years postoperative) experienced GEVs, 3 of which were sufficiently large to require intervention (Figure 3) (42). Kogiso et al. clarified the significantly higher prevalence (33.3%) of esophageal varix in patients with HCC after Fontan procedure compared with post-Fontan patients without HCC (78). As these previous studies show, GEVs can already be present in adolescents, that is, approximately 10 years after the Fontan procedure. Therefore, screening and monitoring of GEVs is encouraged for patients who meet these age or postsurgery milestones.

## 7 PLE

PLE—increased protein loss from the gut—tends to follow a severe, refractory, and chronic course in patients with FALD. Clinical symptoms of PLE include persistent edema, abdominal distention, diarrhea, pleural ascites, and, in severe cases, growth and nutritional disorders and sepsis secondary to hypogammaglobulinemia; however, some cases are asymptomatic except for hypoalbuminemia (53, 79, 80). PLE occurs earlier (i.e., at 3–8 years postoperatively) than liver cirrhosis, HCC, and GEVs, with some cases of PLE occurring only several months after a Fontan operation (53, 81). In Japan, the incidence of PLE after Fontan surgery is 6.2% (82). The 5-year survival rate for patients with Fontan-associated PLE reportedly has improved from 50 to 87% (80, 81). The pathomechanisms of PLE after the Fontan procedure are not fully understood, but several factors are considered to cause an unbalanced lymphatic system, leading to PLE. PLE occurs in the context of advanced FALD (83), whereas the increased in the mesenteric resistance, CVP and pulmonary vascular resistance, low cardiac output, and inflammatory mediators and cells contribute to develop PLE in regardless of the development of liver fibrosis (84–86).

## 8 Evaluation of FALD

### 8.1 Histologic evaluation

Because liver fibrosis is the most characteristic finding in FALD, appropriate assessment of liver fibrosis is necessary for the diagnosis of FALD. In this regard, liver biopsy is the “gold standard” for evaluating liver fibrosis histologically, but various drawbacks such as sampling errors and complications (e.g., bleeding) (87) argue against performing liver biopsy as the routine method for evaluating liver fibrosis in post-Fontan patients, especially pediatric patients. In addition, the lack of an established histologic scoring system for FALD diminishes the relevance of histologic assessment for diagnosing FALD. Conventional histologic assessment methods for liver fibrosis, including the Metavir and Ishak scores and the method of Scheuer, have demonstrated their reliability for the chronic liver disease associated with HCV and HBV (88–91). However, these conventional methods currently are considered inappropriate for assessing the unique histologic features of FALD, including sinusoidal fibrosis and dilation, because those scoring systems focus on liver fibrosis in the portal region. In this regard, Kendall et al. and Wu et al. have improved the Metavir score and developed a new scoring system suitable for Fontan patients (92, 93), and other new assessment schemes such as the congestive hepatic fibrosis score (CHFS) (60) and modified Ishak congestive hepatic fibrosis histologic score (94) have been found to be suitable for this patient population. A previous study comparing the conventional Metavir score and the CHFS for evaluating liver fibrosis in post-Fontan patients clarified that the Metavir score missed 6.7% of cases of sinusoidal fibrosis identified by the CHFS (61). The CHFS (Figures 3a–d) is scored as 0, no fibrosis; 1, central zone fibrosis; 2A, central zone and mild portal fibrosis, with accentuation at the central zone; 2B, at least moderate portal fibrosis with central zone fibrosis and accentuation at the portal zone; 3, bridging fibrosis; and 4, cirrhosis. The CHFS is becoming a widely used scoring scheme for liver histologic assessment in patients who have undergone a Fontan procedure (41, 61, 95).

Regarding the method used to collect the liver tissue, post-Fontan patients tend to present with hepatic congestion, frequently use anticoagulants, and often have low platelet counts. Therefore, conventional ultrasound-guided percutaneous liver biopsy is associated with increased risk of bleeding in these patients, such that transjugular liver biopsy (TJLB) is frequently preferred (41, 94–97). TJLB not only supports liver tissue sampling with a reduced risk of bleeding (87, 95, 97) but also facilitates the acquisition of hemodynamic information [e.g., CVP, hepatic venous pressure gradient (i.e., the difference between the hepatic venous wedge pressure and hepatic venous pressure)] via the concurrent cardiac catheterization (41). However, sometimes post-Fontan patients have curvature or stenosis of the vessels between the jugular and hepatic veins, thus complicating the delivery of the tip of the TJLB cannula to the liver (41). In such cases, ultrasound-guided percutaneous liver biopsy must be used (41). Consequently, for safe and successful liver biopsy and the appropriate interpretation of liver histology in post-Fontan patients, collaboration among cardiologists, hepatologists, interventional radiologists, and histopathologists who understand the characteristics of FALD is critical.

### 8.2 Blood and biochemical examinations

Because liver biopsy is too invasive for the routine screening and monitoring of FALD of asymptomatic post-Fontan patients during long-term follow-up, non-invasive evaluation tools (e.g., serum biomarkers, imaging modalities) have been studied for these populations (64, 98). Blood and biochemical markers sufficiently specific for and relevant to the liver fibrosis of FALD are currently unavailable (64, 98). Given that serum γGTP, AST, and ALT often remain within normal limits regardless of the degree of fibrosis, these markers are not sensitive to the diagnosis of FALD (99, 100). Although the reliability of indirect biochemical markers of fibrosis (e.g., hyaluronic acid, type 4 collagen 7 s, M2BPGi) has been established in the context of chronic hepatitis B and C, these markers fail to accurately predict the degree of liver fibrosis in post-Fontan patients (41). Patients with advanced FALD frequently present with hypoalbuminemia and prolonged PT-INR (9), but because concomitant heart failure and the use of warfarin result in similar laboratory abnormalities, distinguishing FALD from other etiologies solely on the bases of serum albumin and PT-INR values can be difficult. In addition, ascites can result from not only hypoalbuminemia but also from the portal hypertension due to liver cirrhosis as well as from cardiac failure (52, 101). Consequently, in addition to serum albumin, the measurement of plasma BNP or serum NT-proBNP levels is important to clarify the nature of the ascites (102, 103). Although platelet count is not a direct parameter of liver synthetic capacity and fibrosis, high portal pressure increases platelet destruction in the spleen, which results in a decrease in platelet count. It has been reported that extent of the platelet count decline is significantly correlated with the degree of liver fibrosis in FALD patients (104). The MELD-XI score (including bilirubin and creatinine), which excludes the effect of anticoagulants from the MELD score, has also been reported to be significantly associated with liver fibrosis in FALD patients (105). Although these conventional scoring methods may adequately reflect the histological liver fibrosis in FALD patients, there is currently no consensus. Recently, Osakawa et al. used proteome analysis to disclose that serum CD44 concentration is significantly correlated with the degree of liver fibrosis in this population (106). Collaboration between basic science and clinical researchers is anticipated to translate these early findings regarding an FALD-specific marker into future practical applications.

### 8.3 Association between heart conditions and FALD

All patients with Fontan circulation develop FALD over time, but the onset and severity of FALD vary widely. Because patients with various congenital heart diseases who cannot undergo biventricular repair eventually undergo Fontan surgery, their circulatory backgrounds differ greatly, such as differences in endocardial structure and cardiac function, and the specifics of the Fontan surgical procedure can also vary. Recently, it has been reported that heart failure and arrhythmia are risks for the early development of FALD (107), and that valve regurgitation, age, and CVP are risks for cirrhosis and hepatic carcinoma (108, 109). On the other hand, the type of congenital heart disease has not been reported as a risk for developing FALD. In terms of cardiac conditions, it is thought that abnormalities related to a high CVP, such as common atrioventricular valve regurgitation and heart failure, are significantly associated with the risk of developing FALD, and can be predictive of FALD onset. Early intervention to lower CVP prior to the onset of FALD may help slow the onset of FALD.

### 8.4 Imaging modalities

**Figure 4:**
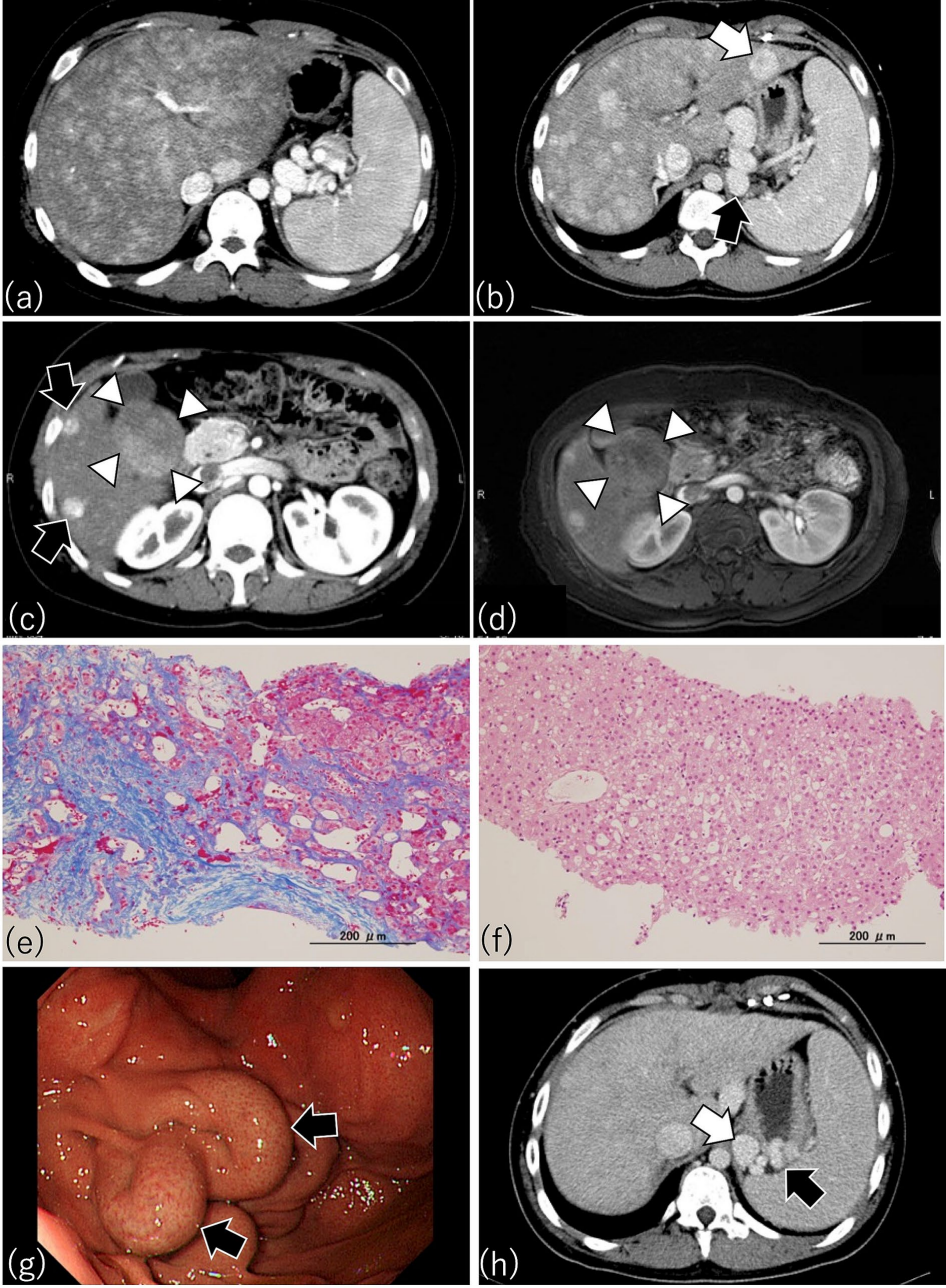
Characteristic findings by CT, MRI, and endoscopy in patients after Fontan procedure. (a) This patient underwent Fontan surgery 17.5 years earlier. Arterial phase imaging of dynamic CT shows diffusely heterogeneous enhancement of the liver, with pronounced peripheral reticular decreased signal caused by venous congestion. The spleen is enlarged, indicating portal hypertension. (b) A patient who had the Fontan procedure 14 years earlier. Dynamic CT shows an enlarged liver with a heterogenous texture and a lesion consistent with focal nodular hyperplasia (FNH) (white arrow). A gastro-renal shunt (black arrow) and enlarged spleen, indicating portal hypertension, are present also. (c) This patient received the Fontan procedure 16.8 years earlier. Arterial phase imaging of dynamic CT shows FNH-like hypervascular lesions (black arrows) and a focal liver lesion (diameter, 5 cm) with a mosaic pattern in S5 and S6 (white arrowheads) (d) Contrast-enhanced MRI with gadolinium ethoxybenzyl diethlenetriamine pentaacetic acid (Gd-EOB-DTPA) of the same patient as in (c) because the mosaic pattern in S5 and S6 suggested hepatocellular carcinoma (HCC). As shown through dynamic CT (c), contrast-enhanced MRI also revealed a mosaic pattern (white arrowheads) suggestive of HCC in the focal liver lesion in S5 and S6; therefore, a targeted liver biopsy was obtained. (e) Azan staining of the biopsy from the liver parenchyma of (c) revealed dilated sinusoids and extensive sinusoidal fibrosis with bridging fibrosis, thus corresponding to a congestive hepatic fibrosis score (CHFS) of 3. (f) H&E staining of the biopsy from the focal liver lesion with the mosaic pattern in (c) disclosed not HCC but regenerative nodular hyperplasia. (g) Esophagogastroscopy in this patient who underwent the Fontan procedure 14 years earlier disclosed an enlarged nodular gastric varix (Lg-*cf*, F2, CW, RC0), thus indicating the need for prophylactic therapy for risk of bleeding. (h) Portal phase imaging with dynamic CT in the same patient as in (g) reveals a gastric varix (black arrow) associated with a gastro-renal shunt (white arrow). The presence of the gastro-renal shunt indicates that balloon-occluded retrograde transvenous obliteration is available as a treatment option for the gastric varix in this patient.

Ultrasonography, liver elastography, CT, and MRI have all been examined for their utility in post-Fontan patients (99, 100, 110–112). Frequent findings on clinical abdominal ultrasonographic images of these patients include hepatomegaly, venous dilatation (due to their elevated CVP), heterogeneous patterns in the liver parenchymal texture, nodular changes at the liver surface, and highly echoic nodules (99, 100, 110). A heterogeneous echogenic parenchymal pattern results not only from congestion but also fibrosis and fatty liver (113), such that using this parameter to estimate histologic changes in post-Fontan patients is difficult. Depending on the degree of liver congestion, veno–venous shunts between hepatic veins can be appreciated via ultrasonography. CT and MRI reveal hepatic fibrosis as heterogeneous parenchymal enhancement with a mosaic or reticular pattern (Figure 4a) (111). In addition, liver fibrosis on T2- or diffusion-weighted MRI appears as hyperintense mottled areas along the organ periphery (112). Contrast MRI scans demonstrate a reticular, patchy enhancement pattern in both the portal venous and delayed phases (114). Patients who have undergone a Fontan procedure are prone to develop a variety of benign or malignant focal liver lesions, including FNH, NRH, and HCC, all of which can be discerned or suspected by using ultrasonography, CT, or MRI (Figures 4b–f) (42, 111, 115, 116). Those imaging features are helpful in the assessment of FALD and facilitate decision-making regarding further examination (e.g., liver biopsy) and treatment strategies. Therefore, collaboration with imaging and interventional radiologists and hepatologists familiar with the unique imaging features of FALD is key.

Regarding other non-invasive and non-radiologic modalities for assessing liver fibrosis in post-Fontan patients, several recent studies have assessed whether the liver stiffness measured via ultrasound elastography (i.e., share wave elastography, transient elastography) or MR elastography reflects the degree of liver fibrosis in FALD (41, 94, 97, 117–119). Elastography-based liver stiffness is an established screening tool for liver fibrosis and cirrhosis in pediatric and adult patients with HCV, HBV, or NAFLD (120–124). However, for patients with FALD, the hepatic congestion due to increased CVP and portal venous pressure artifactually increases the liver stiffness measured via transient elastography, thus hampering the use of this modality for the accurate interpretation of liver stiffness as a parameter of liver fibrosis (41, 97, 125). On the other hand, a previous study demonstrated that liver and spleen stiffness were useful indicators of significant liver fibrosis and portal hypertension (126). Similarly, shear wave elastography and MR elastography have been shown to poorly predict the severity of liver fibrosis in patients with FALD (118, 119); in particular, venous congestion impairs MR elastography-based assessment of liver stiffness (94, 118). Therefore, although elastography may be able to assess portal hypertension with reasonable accuracy, its use as a sole technique has limitations regarding measuring the degree of liver fibrosis in post-Fontan patients. However, several studies suggest significant correlation between the increase in liver stiffness and the time since Fontan surgery (41, 99, 110). In addition, findings on MR elastography were reflective of Fontan-associated circulatory failure in pediatric and adult post-Fontan patients (127, 128). This circulatory failure typically leads to the development of hemodynamic and cardiovascular complications (e.g., high CVP, regurgitation through the atrioventricular cardiac valves, supraventricular tachycardia) during long-term follow-up. These conditions also contribute to the development of FALD. In this way, the relationship between liver stiffness and the time since Fontan surgery may reflect the complex conditions resulting from liver fibrosis and the hemodynamic and cardiovascular insults due to Fontan-associated circulatory failure. Furthermore, a recent study clarified that the liver stiffness measured through transient elastography is independent of food intake and breathing movements and therefore can be performed on young post-Fontan patients (129). Therefore, liver stiffness can (and perhaps should) be measured as part of a routine workup, to provide physicians information regarding the deteriorating hemodynamic and cardiovascular conditions in patients who have undergone Fontan surgery.

### 8.5 Evaluation of HCC, GEVs, and PLE

Alpha fetoprotein (AFP) is a common blood biochemical marker for HCC. However, in one study, an AFP level above 400 ng/mL was considered diagnostic of HCC in post-Fontan patients (130), whereas other studies reported that 21.7–26% of HCC cases in post-Fontan patients showed normal AFP values (73, 76). These conflicting data thus call into question the significance of AFP as a marker of HCC in this population. In addition, the significance of lectin-bound AFP (AFP-L3%) for HCC in those patients has not yet been evaluated and therefore is unclear. Another blood biochemical marker of HCC, PIVKA-II, is useless in post-Fontan patients because many of them receive the anticoagulant warfarin, which elevates PIVKA-II levels regardless of the presence of HCC. Alternatively, the combination of polysplenia and a high MELD-X1 score may predict the development of HCC in post-Fontan patients (78).

Because the risk of developing HCC increases rapidly after 10 years postoperatively, imaging modalities including ultrasonography, contrast CT, and MRI are necessary during the long-term follow-up of post-Fontan patients so that HCC will not be missed. However, options regarding imaging modality available for patients with pacemakers are limited. To further assess whether focal liver lesions are HCC, contrast-enhanced MRI using gadolinium ethoxybenzyl diethlenetriamine pentaacetic acid (Gd-EOB-DTPA) or contrast-enhanced ultrasonography with sonazoid are valuable (131–133). The time course of the Gd-EOB-DTPA-enhanced MRI signal intensity in HCC of FALD differs from that of FNH in FALD and of HCC in chronic hepatitis C (131). In contrast-enhanced ultrasonography, poorly differentiated HCC of FALD shows homogeneous enhancement during the arterial-dominant phase and progressive hypoechogenicity relative to the adjacent liver parenchyma during the portal-dominant phase; in contrast, a regenerative nodule is an isoechoic mass relative to the adjacent liver parenchyma during the portal-dominant phase (133). However, despite the use of Gd-EOB-DTPA-enhanced MRI, differentiating HCC from FNH or NRH in the livers of post-Fontan patients can remain difficult (Figures 4c,d) (111). In these cases, ultrasound-guided percutaneous targeted biopsy of a focal liver lesion may be necessary to exclude the possibility of HCC (Figures 4c–f). Because the diagnosis of HCC in post-Fontan patients requires careful interpretation of several imaging modalities and (sometimes) histologic confirmation, appropriate assessment of focal liver lesions in this population requires collaboration among radiologists, hepatologists, and histopathologists.

**Figure 5:**
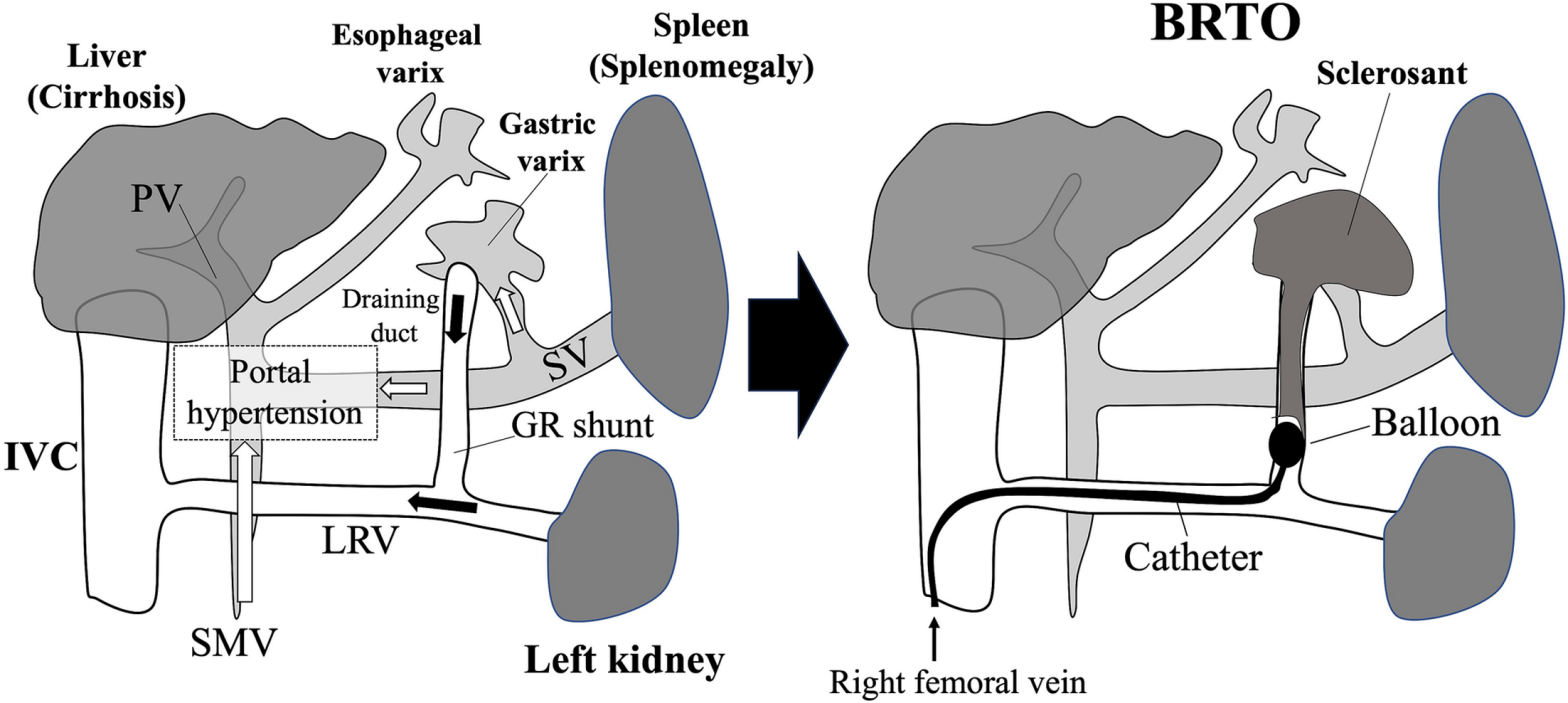
Gastroesophageal varices (GEVs) in patients who received the Fontan procedure and balloon-occluded retrograde transvenous obliteration (BRTO) for treatment of gastric varix. The tip of the balloon catheter is placed in the draining duct of the gastric varix via the gastro-renal shunt. Blood flow in the gastric varix is regulated by occluding the draining duct with a balloon, and then a sclerosing agent is injected. Microcoils are sometimes placed also. GR shunt, gastro-renal shunt; IVC, inferior vena cava; LRV, left renal vein; PV, portal vein; SMV, superior mesenteric vein; SV, splenic vein.

GEVs are often missed because they typically are asymptomatic until they rupture. Because their rupture can be life-threatening, screening for and monitoring of GEVs are important during the long-term follow-up of post-Fontan patients. Even adolescent and young-adult patients who were an average of 12 years after Fontan surgery developed enlarged GEVs at risk of rupture (42). Therefore, when post-Fontan patients become adolescents or when they are 10 years after surgery, they are recommended to receive screening for GEVs. Duodenogastroscopy is the gold-standard screening method for GEVs (Figure 4g); abdominal dynamic CT examination is another option (42). In addition to its utility for evaluating GEVs, abdominal dynamic CT can provide information on the draining collateral veins (e.g., gastro-renal and spleno-renal shunts) frequently found in post-Fontan patients (Figure 4h). Furthermore, because a gastro-renal shunt typically is used as a therapeutic route for balloon-occluded retrograde transvenous obliteration (BRTO) of enlarged GEVs with a high risk of rupture (Figure 5) (42), abdominal dynamic CT is useful for both screening of GEVs and for determining therapeutic interventions. When abdominal dynamic CT indicates the presence of a GEV, duodenogastroscopy is encouraged to confirm this finding. Some physicians hesitate to perform both duodenogastroscopy and abdominal dynamic CT in asymptomatic patients, but a previous study demonstrated that a platelet count below 119 × 10^9^/L may prompt the use of abdominal dynamic CT to identify GEVs (42). Given the features and risks of GEVs, their successful management requires cooperation among pediatric and adult hepatogastroenterologists as well as imaging and interventional radiologists.

Regarding PLE as a component of FALD, hypoalbuminemia is the most definitive laboratory feature. To diagnose PLE, other causes of hypoalbuminemia, such as renal or hepatic disease and infectious gastroenteritis, must be ruled out first. Subsequent steps in the diagnosis of PLE include measuring fecal levels of alpha-1-antitrypsin (A1AT) (spot, >54 mg/dL; A1AT clearance, >27 mL/24 h without diarrhea, >56 mL/24 h with diarrhea) (80, 134) and performing nuclear scintigraphy (80, 134–136).

**Figure 6:**
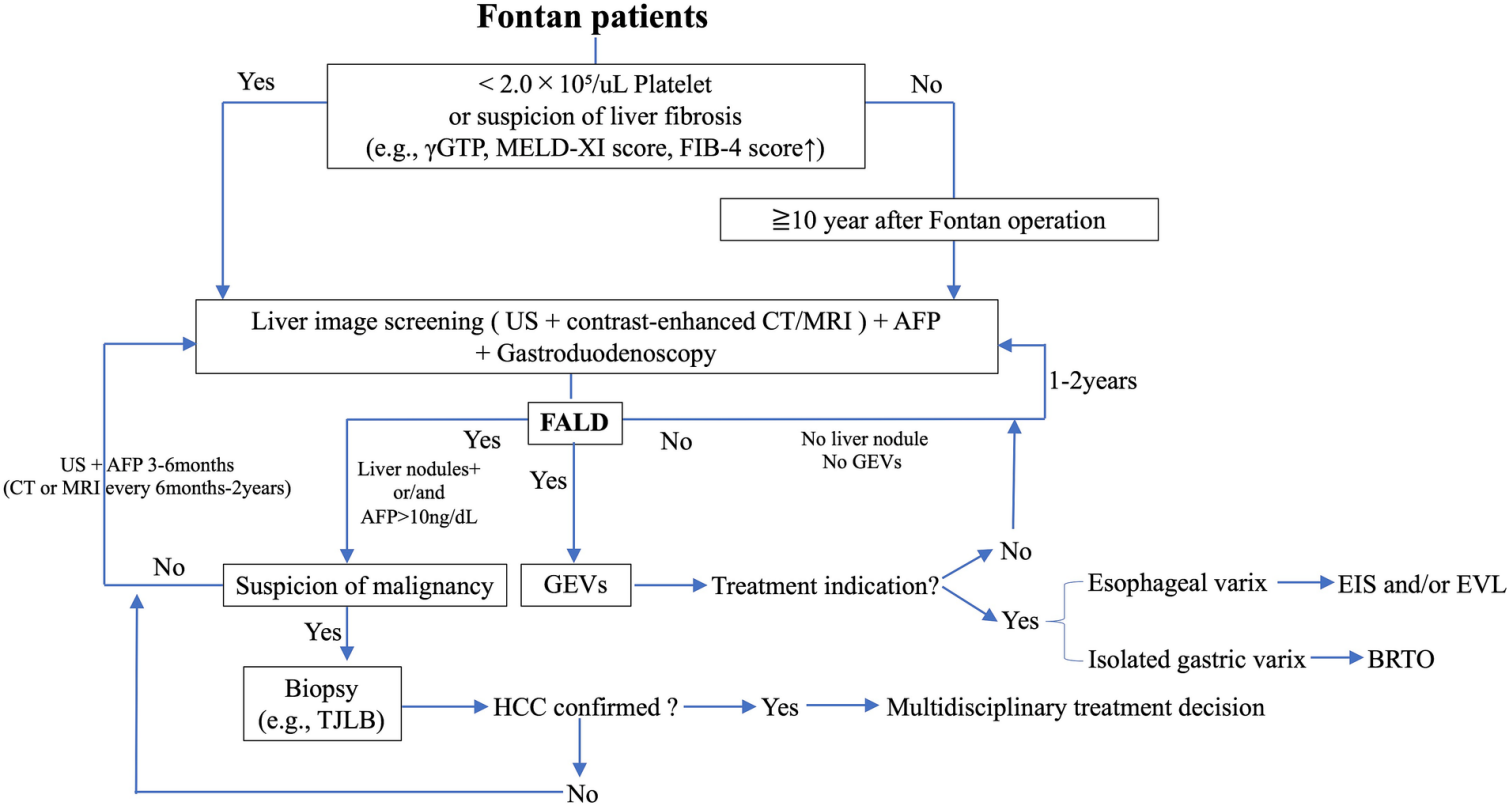
Flow diagram for the surveillance of liver nodules, HCC, and GEVs in FALD. AFP, alpha fetoprotein; BRTO, balloon-occluded retrograde transvenous obliteration; CT, computed tomography; EIS, endoscopic injection sclerotherapy; EVL, endoscopic variceal ligation; FALD, Fontan-associated liver disease; GEVs, gastroesophageal varices; *γ*GTP, γ-glutamyl transpeptidase; HCC, hepatocellular carcinoma; MRI, magnetic resonance imaging; TJLB, transjugular liver biopsy.

Although it is difficult to build an appropriate guideline for the evaluation and diagnosis of FALD because of a lack of accumulated evidence, we have constructed an algorithm for the diagnosis and follow-up of FALD focusing on liver nodules, HCC, and GEVs (Figure 6).

## 9 Treatment of FALD and the importance of multidisciplinary collaboration

Currently there is no established treatment for FALD. Because the liver fibrosis after Fontan surgery develops due to intrahepatic hypoxia, thrombus formation, and elevated sinusoidal stiffness, medical (e.g., diuretics, ACE inhibitor, warfarin) and surgical (e.g., valve repair, pacemaker placement) management and catheter-based interventions (e.g., occlusion of collaterals, creation of fenestration) to counter Fontan circulation contribute to preventing the development of FALD. In addition, pulmonary vasodilators are described to reduce pulmonary artery resistance and increase vascular compliance, pulmonary artery diameter, and cardiac output in Fontan patients (137), thus those pulmonary vasodilators are also included in medications to prevent FALD. In terms of the specific treatment for each hepato-gastrointestinal complication (e.g., HCC, GEVs, PLE), HCC in patients with FALD has been treated through transcatheter arterial chemoembolization, hepatic arterial infusion chemotherapy, radiofrequency ablation, proton beam therapy, and surgical resection (51, 76, 132). Although the HCC treatment options used depend on a patient’s liver function and the number and size of the tumors, a systematic review including 65 biopsy-proven HCCs in patients with FALD demonstrated that transcatheter arterial chemoembolization was the most common intervention (76). Surgical resection of HCC in post-Fontan patients has been reported (51, 76, 138) but is not commonly performed in light of patients’ reduced liver function, the presence of portal hypertension, and the high cardiovascular impact of surgery. According to guidelines from EASL and the American Association for the Study of Liver Disease, portal hypertension is a relative contraindication to hepatic resection because of the high risk of postoperative liver decompensation (139, 140). Therefore, early detection of HCC is important to support prompt, conservative therapeutic intervention for post-Fontan patients with FALD. Although there is no consensus regarding preventive therapy for HCC, a recent study disclosed that the lack of warfarin treatment and situs inversus were associated with HCC development after Fontan surgery (141). Because thrombus formation is involved in the pathomechanism of liver fibrogenesis, which is the first stage in the development of HCC (71), warfarin treatment is anticipated to prevent HCC development, and this expectation should be clarified through further study. Collaboration among experienced interventional radiologists, hepatologists, oncologists, and surgeons is necessary to select and accomplish appropriate medical, radiologic, and surgical treatments for HCC in post-Fontan patients.

**Figure 7:**
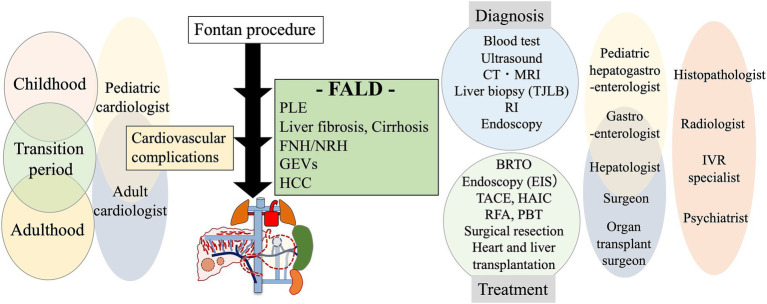
A multidisciplinary collaborative approach to the management of FALD. CT, computed tomography; FALD, Fontan-associated liver disease; FNH, focal nodular hyperplasia; GEVs, gastroesophageal varices; HAIC, hepatic arterial infusion chemotherapy; HCC, hepatocellular carcinoma; MRI, magnetic resonance imaging; NRH, nodular regenerative hyperplasia; PBT, proton beam therapy; PLE, protein-losing enteropathy; RFA, radiofrequency ablation; RI, radio isotope; TACE, transcatheter arterial chemoembolization; TJLB, transjugular liver biopsy.

Similar to the need for multidisciplinary management of liver fibrosis and tumors, collaborative care in the treatment of GEVs due to portal hypertension is important to improve the quality of life of post-Fontan patients. Our previous study revealed that adolescent post-Fontan patients have a risk of enlarged GEVs, which require bleeding prophylaxis via an interventional radiology approach (42). Whereas esophageal varices generally are addressed through endoscopic means, such as endoscopic sclerotherapy and ligation, GEVs are treated through endoscopic procedures or BRTO depending on the presence of gastro-renal shunts (Figure 5). The BRTO technique was developed in Japan and has been applied to GEVs and portosystemic shunts in post-Fontan patients (Figure 5) (42, 142). In BRTO, a gastro-renal shunt can be used to access target GEVs (Figure 5) (143). In cases with a gastro-renal shunt, BRTO is a preferable therapeutic approach because it, unlike endoscopic sclerotherapy, lacks the risk of leakage of the sclerosing agent into the systemic circulation, leading to pulmonary embolization. An intraoperative shunt occlusion test should be performed during BRTO in post-Fontan patients to measure the venous pressure at the time of shunt occlusion; given that post-Fontan GEVs arise due to portal hypertension, BRTO may exacerbate the increased portal pressure, thus it is recommended to evaluate the IVC pressure by the preliminary transient balloon occlusion test. Otherwise, Fontan track stenting (conduit or pulmonary branches) may be considered to improve the IVC compliance. In addition, because BRTO carries a risk of postoperative development of esophageal varices (144), endoscopic follow-up is recommended. Therefore, treatment plans regarding GEVs should be decided through collaboration including gastrointestinal endoscopists and interventional radiologists according to careful assessment of abdominal dynamic CT images obtained by imaging radiologists; endoscopic follow-up requires input from gastrointestinal endoscopists and pediatric hepatogastroenterologists (Figure 7).

The medical treatment of PLE in post-Fontan patients typically involves a combination of diuretics, digitalis, afterload reduction (e.g., ACE inhibitor), low-fat diet and MCT oil, and intermittent infusion of albumin and gamma globulins (53, 81). Oral and intravenous corticosteroids have shown benefit for treating PLE in post-Fontan patients (53, 86, 145), but some cases are refractory (53). Because PLE tends to relapse after the discontinuation of corticosteroids, maintenance therapy using low-dose oral budesonide has been described (146). The mechanism underlying the usefulness of corticosteroids to counter PLE is not fully understood, but circulating inflammatory mediators and inflammatory cell infiltration are speculated to be involved in the development of PLE and thus may be amenable to regulation by corticosteroids (85, 86, 145). Surgical and medical cardiac interventions, Fontan fenestration, obstruction relief, ablation therapy, and valve replacement have all been demonstrated to ameliorate PLE (53, 81). In addition, because an unbalanced lymphatic system is involved in the etiology of PLE, surgical decompression of the thoracic duct and percutaneous lymphatic embolization can be effective (147–150). As for other components of FALD, deciding on an appropriate treatment course for PLE in post-Fontan patients requires discussion among cardiologists, hepatogastroenterologists, interventional radiologists, and surgeons (Figure 7).

The definitive treatment for patients with Fontan circulation is heart transplantation, and the number of patients with Fontan circulation eligible for transplantation is increasing as survival rates improve (151, 152). Also, the incidence of liver disease associated with Fontan circulation has decreased (153), suggesting that liver disease may improve after heart transplantation (154). In terms of end-stage FALD, although organ transplantation is the definitive treatment, transplantation of the liver only is not recommended (155). Patients with FALD have increased CVP and low cardiac output, and the liver transplantation procedure will further stress their cardiac function and worsen heart failure symptoms. For this reason, combined heart–liver transplantation or sequential heart transplantation and liver transplantation are considered appropriate treatment options for end-stage FALD (52, 153). The multidisciplinary care team for patients with end-stage FALD therefore should include liver and heart transplant surgeons, cardiologists, and hepatologists (Figure 7). A comparison of the results of isolated heart transplantation versus combined heart–liver transplantation in patients with congenital heart disease showed there to be no difference in mortality at postoperative day 30 or 1 year (156). Furthermore, there are also significant differences in outcomes between combined heart–liver transplantation and isolated heart transplantation in Fontan patients, despite the combined heart–liver transplantation generally being selected in older patients with advanced FALD (157, 158). Interestingly, a comparison of isolated-heart-transplantation and combined heart–liver transplantation in Fontan patients showed significantly less graft failure in the combined heart–liver transplantation group. This may be due to immune tolerance in the transplanted liver reducing rejection (159–161) but further studies are warranted. Currently, there are no clear guidelines for determining the need for an isolated heart transplantation versus a combined heart–liver transplantation, mainly because few studies have been conducted on transplant outcomes and the surgical criteria vary from transplant center to transplant center; therefore, the timing and selection of patients should be carefully determined based on the scores and surrogate markers reported to predict mortality after transplantation. In this regard, MELD-11 score (162), serum bilirubin level (163), time from Fontan failure to evaluation, NYHA class IV, varicose veins, and arteriovenous collaterals have all been reported (164), and evaluation of FALD before transplantation showing cirrhosis, varices, splenomegaly, and ≥ 2 paracenteses is correlated with survival after combined heart–liver transplantation or isolated heart transplantation (159). Thus, evaluation of FALD is necessary for effective selection of patients requiring combined heart–liver transplantation, and accurate scoring of FALD will be necessary with the expected increase of Fontan circulation transplant cases in the future.

Other important issues to address in the care of post-Fontan patients are their mental health and need for transitional care. Post-Fontan patients are exposed to severe mental health challenges, with multiple surgeries and repeated hospitalizations and a high incidence of complications despite their young age. Indeed, this population has a higher probability of psychiatric diagnosis (65%) than their healthy peers (22%) (165). Post-Fontan patients receive considerable physical and mental support from their parents during childhood, but the contribution of parental care diminishes as these patients progress to adulthood. Consequently, many post-Fontan patients may discontinue follow-up after they transfer from the pediatric cardiology service to adult care units (166). The discontinuation of hospital visits caused by insufficient mental health support will disturb FALD surveillance and delay intervention in FALD patients. Providing them appropriate and sustainable care that addresses their mental health is important for effective surveillance and appropriate intervention in FALD, therefore a multidisciplinary, collaborative team that includes psychiatrists and psychologists as well as social services support from local welfare departments and health programs is required (Figure 7).

## 10 Conclusion

Comprising a variety of hepato-gastrointestinal complications including liver fibrosis, HCC, GEVs, and PLE, FALD frequently arises during long-term follow-up after the Fontan operation. Many issues remain regarding the underlying pathomechanisms of FALD, reliable diagnostic methods, and effective preventive and therapeutic strategies. Although cardiovascular complications after the Fontan procedure can be managed successfully by pediatric and adult cardiologists, managing FALD requires both appropriate diagnostic work and treatment by a multidisciplinary team composed of pediatric and adult cardiologists, pediatric and adult gastroenterologists and liver specialists, imaging and interventional radiologists, thoracic surgeons, histopathologists, and liver and heart transplant surgeons. To support the transition of post-Fontan patients from pediatric to adult care units and to address their mental health needs, the inclusion of psychiatrists and other mental health care providers is encouraged. Finally, collaboration between clinical specialists and basic scientists will further the elucidation of FALD pathomechanisms and the development of FALD-specific biomarkers.

## Glossary

A1AT: alpha-1-antitrypsin
ACE: angiotensin-converting enzyme
AFP: alpha fetoprotein
ALT: alanine aminotransferase
APRI: AST to platelet ratio index
AST: aspartate aminotransferase
BNP: brain natriuretic hormone
BRTO: balloon-occluded retrograde transvenous obliteration
CHFS: congestive hepatic fibrosis score
CT: computed tomography
CVP: central venous pressure
EASL: European Association for the Study of the Liver
ERN-RARE-LIVER: European Reference Network on Rare Liver Diseases
FALD: Fontan-associated liver disease
FIB-4: fibrosis-4
FNH: focal nodular hyperplasia
Gd-EOB-DTPA: gadolinium ethoxybenzyl diethlenetriamine pentaacetic acid
GEVs: gastroesophageal varices
*γ*GTP: γ-glutamyl transpeptidase
HBV: hepatitis B virus
HCC: hepatocellular carcinoma
HCV: hepatitis C virus
M2BPGi: Mac-2 binding protein glycosylation isomer
MELD: model for end-stage liver disease
MR elastography: magnetic resonance elastography
MCT: medium chain triglyceride
MRI: magnetic resonance imaging
NAFLD: non-alcoholic fatty liver disease
NRH: nodular regenerative hyperplasia
PIVKA-II: protein induced by vitamin K absence or antagonist-II
PLE: protein losing enteropathy
PT-INR: prothrombin time-international normalized ratio
TCPC: total cavopulmonary connection
TJLB: transjugular liver biopsy
